# Folate Transporters in Placentas from Preterm Newborns and Their Relation to Cord Blood Folate and Vitamin B12 Levels

**DOI:** 10.1371/journal.pone.0170389

**Published:** 2017-01-19

**Authors:** Erika Castaño, Lorena Caviedes, Sandra Hirsch, Miguel Llanos, Germán Iñiguez, Ana María Ronco

**Affiliations:** 1 Laboratory of Nutrition and Metabolic Regulation, Human Nutrition Unit, Institute of Nutrition and Food Technology, Dr. Fernando Monckeberg Barros (INTA), University of Chile, Santiago, Chile; 2 Mother and Child Research Institute, Division of Medical Sciences, School of Medicine, University of Chile, Santiago, Chile; Xavier Bichat Medical School, INSERM-CNRS - Université Paris Diderot, FRANCE

## Abstract

Folate deficiency during pregnancy has been related to low birth weight, preterm (PT) birth and other health risks in the offspring; however, it is unknown whether prematurity is related to low folate transport through the placenta due to altered expression of specific folate transporters. We determined placental expression (mRNA and protein concentrations by RT-qPCR and WB respectively) of specific folate transporters: RFC, PCFT/HCP1 and FOLR1 in chorionic (fetal) and basal (maternal) plates of placentas of PT pregnancies (PT, 32–36 weeks, n = 51). Term placentas were used as controls (T, 37–41 weeks, n = 47). Folates and vitamin B12 levels were measured by electrochemiluminescence in umbilical cord blood of newborns. FOLR1 mRNA expression was lower and protein concentration higher in PT placentas (both plates) relative to the control group (p <0.05). In addition, gestational age was positively correlated with mRNA expression (Rho = 0.7), and negatively with protein concentration (Rho = -0.7 for chorionic and -0.43 for basal plate). PCFT/HCP1 mRNA was lower in PT placentas, without changes in protein levels. RFC did not differ in PT placentas compared to controls. PT newborns presented higher cord blood folate level (p = 0.049) along with lower vitamin B12 concentration compared to controls (p = 0.037).In conclusion, placental FOLR1 mRNA was positively associated with gestational age. Conversely, FOLR1 protein concentrations along with folate/vitamin B12 ratio in cord blood were negatively associated with gestational age. Placental FOLR1 is likely the main placental folate transporter to the fetus in newborns.

## Introduction

Folates are water-soluble vitamins of the B complex that are naturally present in foods as reduced forms or glutamate chains. The synthetic form, folic acid (FA), is fully oxidized and more stable; it is frequently used in fortified foods or as supplements [1,2]. Folates are needed for fetal growth and placental development, since they activate cell growth and biosynthetic processes that are essential during pregnancy [3]. For these reasons maternal requirements during pregnancy are 50% greater than adult requirements (400 μg/day) [3,4].

Folate deficiency has been associated with preterm births (PT) [1], defined as <37 week gestation at birth; these are a major public health concern [5], with reported rates ranging from 5% to 20% worldwide [6]. Deficient maternal folate intake [1] and decreases in folate bioavailability associated with reduced folate transport to the fetus or the presence of FRα (folate receptor alpha) autoantibodies (FRAbs) [7] are included in the etiology of PT. Also, folate deficiency may induce low birth weight and other altered birth outcomes such as neural tube defects (NTD) [3,4,8]. Folates and FA reduce the risk of NTD [8], thus several countries have implemented food fortification programs with FA to increase the consumption of FA during pregnancy [9]. Chile started FA fortification in 2001 with 2.4 mg of FA/1000 g in wheat flour [10], which led to a 43% decrease in the prevalence of NTD two years later [11]. This greater supply of FA along with the greater bioavailability of FA (near 100%), twice that of natural folate [4], have dramatically increased FA consumption levels with unknown long-term effects on offspring. It is important to note that FA has a tolerable upper intake limit (UL) value of 1000 μg/day [4], mainly because higher levels may mask vitamin B12 deficiency [12,13]. Vitamin B12, which is present mainly in foods of animal origin [14], is also essential during pregnancy due to its role in folate metabolism, normal cell growth, prevention of birth defects and neurocognitive development [15,16]. The maternal requirement of vitamin B12 during pregnancy is slightly higher than in adults (from 2.4 to 2.6 μg/day) [4].

Due to the key role played by these vitamins (folates and vitamin B12) in the metabolism of methionine and methylation reactions, a deficiency, excess or imbalance in the supply of some of them during pregnancy could create an adverse intrauterine environment, and consequently induce fetal programming through epigenetic mechanisms [17,18]. Some studies have reported lower fetal growth due to a high folate/vitamin B12 ratio [19]. However, more studies are needed to confirm these findings.

The delivery of nutrients from mother to fetus only occurs through the placenta [20]. Three specific folate transport mechanisms operate in the human placenta, including folate receptor (FOLR) in its -α (FOLR1) and β (FOLR2) forms [21], the reduced folate carrier (RFC) and the proton-coupled folate transporter (PCFT/HCP1) [22–24]. The transfer of folate from maternal circulation to the fetus is the result of the coordinated action of these transporters, although there is published evidence that FRα is the primary transporter for maternal folate to the fetus [25]. Involvement of members of the ABC superfamily of transporters in FA cellular homeostasis in the human placenta has also been reported [23].

Since altered folate levels have been linked to preterm pregnancies, the aim of this study was to investigate whether preterm conditions present in shorter gestational periods (< 37 weeks) are related to decreased transport of folates to the fetus due to an altered expression of placental folate transporters.

## Materials and Methods

### Sample collection

The experimental design corresponds to a quasi-experimental study performed separately in samples of the maternal side (basal plate: BP) and fetal side (chorionic plate: CP) of placentas of healthy moderate PT pregnancies (32–36 weeks gestation, n = 47) and term pregnancies (T, 37–41 weeks) with birth weights adequate for gestational age, categorized by current curves for the Chilean population [26]. Term placentas were used as the control group.

Exclusion criteria included pregnancies with <32 weeks gestation, diabetes, hypertension, preeclampsia, smoking (consumption >5 cigarettes/day), alcoholism or drug use, twin or multiple pregnancies and genetic diseases in the newborn. The Scientific Ethics Committee of the Central Metropolitan Health Service, the Ethics Committee of the Faculty of Medicine and INTA, University of Chile, approved the study. The mothers in this study signed an informed consent.

### Placental explants

We extracted explants from placentas (10 mg) obtained between 2010 and 2014 in the San Borja Arriarán Hospital in southern Santiago, Chile. We selected two groups of placentas: term pregnancies, n = 47 and preterm pregnancies n = 51. We extracted explants of rectangular pieces (approximately 7x5cm, 0.5cm^3^) from placentas after a cross-section near the umbilical cord, differentiating the CP and BP. The pieces were washed with cold saline to remove excess blood and were individually frozen in cryotubes at -80°C until analysis.

### RNA extraction, cDNA synthesis and real-time PCR

Total RNA from both plates of placental tissues was extracted with RNAeasy kit columns (Lipid Tissue Mini Kit Qiagen) according to manufacturer's instructions, and frozen in aliquots at -80°C until cDNA synthesis. The RNA integrity of each sample was evaluated by a 260/280 ratio of 1.7–1.9 and by electrophoresis in agarose gels. One microgram of total mRNA was used for the synthesis of cDNA with the M-MLV reverse transcriptase kit (RT, Promega, Wisconsin, USA) following manufacturer’s instructions. For mRNA quantification of RFC, PCFT/HCP1 and FOLR1 we used the Eco qPCR System (Illumina San Diego, CA, U.S.A). Results were analyzed by Eco Real-time PCR System Software v4.1 (Illumina) and calculated by the -2^ΔΔ^ method [27] following the MIQE Guidelines [28]. Results were expressed in relation to the geometric mean expression of three of the most stable housekeeping genes (GAPDH, YWHAZ and β-actin) [29], using the term group as control. Gene primers were described previously [30]. Life Technologies supplied Primers for FOLR1 (assay code: Hs01124177_m1).

### Protein extraction and western blot

Proteins of placental explants of both plates (CP and BP) from both groups were extracted with lysis buffer (Bio Source, Invitrogen) supplemented with Triton X-100 detergent and 1% protease inhibitor cocktail (Roche) and frozen at -80°C until Western blotting. Protein concentrations were determined by the Pierce ® BCA Protein Kit Assay (Thermo Scientific, USA) using a standard curve of fetal bovine albumin (BSA 1000 mg/mL). Thirty μg of total proteins were used for RFC (140kDa) and HCP1/ PCF (55kDa); and 40 μg for FOLR1 (37-42kDa); which were mixed with loading buffer (AccuRuler RGB PLUS Prestained Protein Ladder) in a 1:1 ratio and subsequently separated by electrophoresis in polyacrylamide gels (Tris-glycine SDS-polyacrylamide 8%). We used two loading controls depending on the MW of each protein: β-Actin 42kDa or Vinculin 100 – 127kDa. The proteins were transferred to a PVDF membrane (0,2μm) at 60mA overnight.

The membranes were blocked with 5% skim milk (in Tween-TBS 1X) for 1 h, washed with T-TBS 1X and incubated with specific antibodies against each folate transporter. As primary antibody, blots were incubated with anti FOLR1 (diluted 1:1000, R&D Systems–AF5646) and subsequently incubated with an anti-goat antibody (diluted 1:5000, R&D Systems—HAF019). For PCFT/HCP1 blots were first incubated with an antibody (diluted 1:7000, Abcam Inc. ab98043) and subsequently with a second anti-rabbit antibody (diluted 1:500, Rockland Antibodies & Assays– 611–1302), which also was used as second antibody for the detection of RFC (first antibody from Abcam Inc. ab3853, diluted 1:7000). For controls we used anti β-actin (Sigma-Aldrich–A1978, diluted 1:1 million,) or anti-vinculin (Santa Cruz Biotechnology Inc. sc-25336, diluted 1:6000), depending on the molecular weight of folate transporters, which were incubated for 2 h at room temperature under stirring. Blots were then incubated with a second anti-mouse antibody (Rockland Antibodies & assays– 610–1319 diluted 1:20000) for 1 h at room temperature under stirring.

The blots were visualized and quantified with Westar reagent chemiluminescent Supernova η Substrate for Western Blot Kit 100mL Cyanagen using a 510 BioSpectrum Multispectral Imaging System and quantified by UVP Framework in the WorksLS Vision software.

### Analysis of folates and vitamin B12 levels in cord blood serum

Blood samples obtained from umbilical cords at birth were collected in sterile red cap serum tubes (without additives). Blood samples were transported within 20 minutes of sampling to the laboratory in special containers on ice, protected from light and immediately centrifuged at 3000 g for 10 min at 4°C. The serum was collected, aliquoted in polypropylene tubes and stored at −80°C. Folates and vitamin B12 concentrations were determined by electrochemiluminescence in an automated Cobas Team 411 (Roche) using Roche reagents. The analyses were performed in a clinical laboratory (Vida Integra) within international standards established for these analyses, with 58 registrations of accredited institutional providers. Inter-assay and intra-assay coefficient variations were 11.8% and 3%, respectively for folate and 10.2% and 1.6% for vitamin B12.

### Statistical analysis

To assess the normality of variables we used the Shapiro Wilk test. To compare differences between groups we used the nonparametric Mann Whitney test. For correlation analyses, we used Spearman correlation. All results are expressed as median (25^th^– 75^th^ percentiles). Statistical analysis was performed using STATA statistical software, version 13 for Mac. A p <0.05 was considered significant.

## Results

### Sample description

The anthropometric characteristics of the newborns whose placenta samples were analyzed in the study are presented in **Table 1**. As expected, birth weight and height were lower in PT compared to term newborns.

**Table 1:** Characteristics of mothers and newborns.

Variable	T (n = 47)	PT (n = 51)
Maternal age (years)	27 (22–33)	28 (21–32)
Maternal BMI (Kg/m^2^)	25 (21–28)	28 (25–30)
Gestational age (weeks)	39 (37–40)	35 (34–36)*
Gender male/female^&^	22/22	23/19
Birth weight (g)	3430 (3120–3710)	2200 (1890–2440)*
Weight z score (SD)	-0.2 (-0.6–0.8)	-1.2 (-1.74 –-0.54)*
Length (cm)	50 (49–51)	45 (42–46)*
Length z-score (SD)	-0.1 (-0.7–0.5)	-1.1 (-1.8–0.3)*
Placental weight (g)	604 (563–700)	513 (412–600)#

T: term newborns; PT: preterm newborns. Data are shown as median (25–75^th^ percentiles)

^&^Absolute number

*p<0.0001 and

#p = 0.0003 compared to term newborns, Mann Whitney test.

### mRNA expression of folate transporters

**Fig 1:**
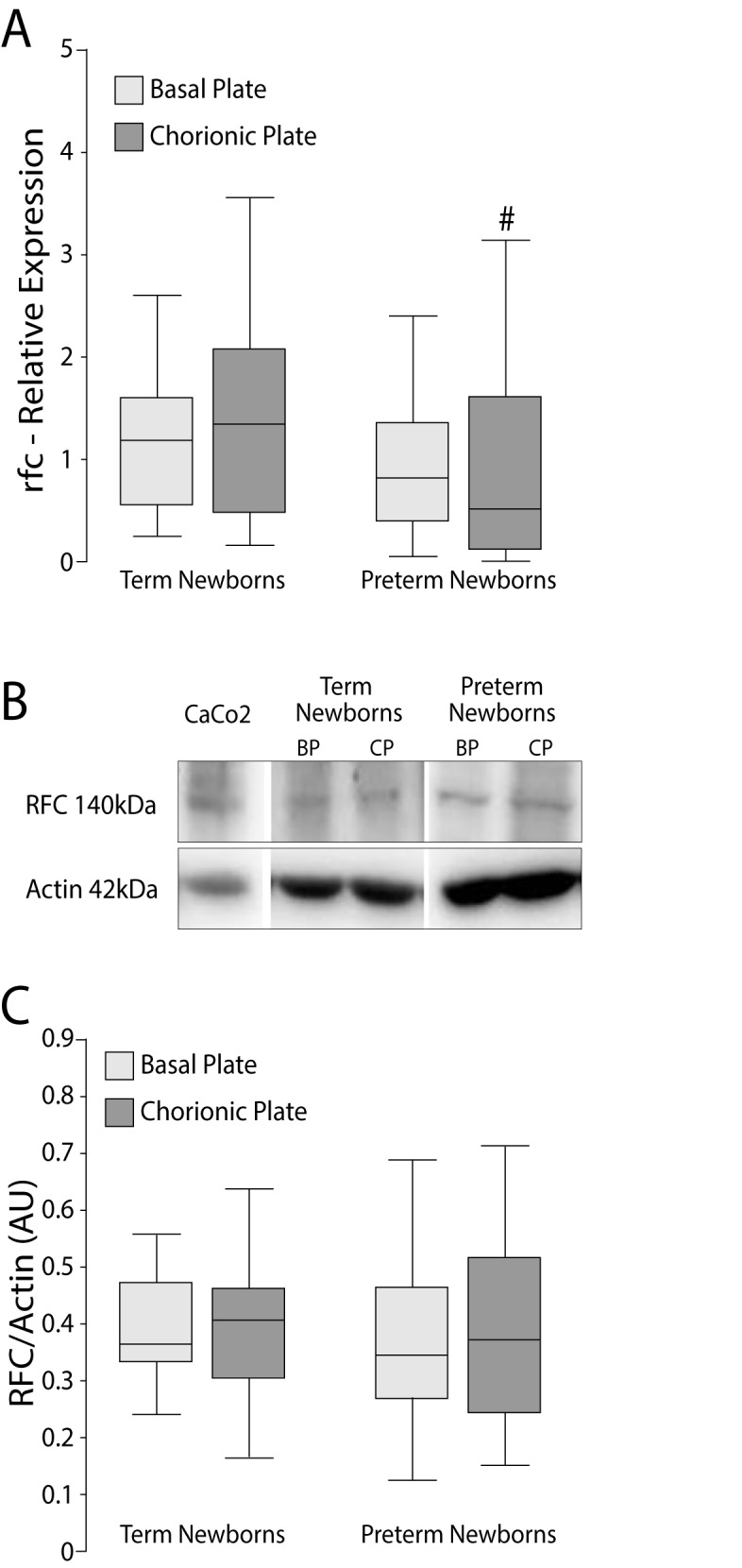
Relative expression (mRNA) and protein levels of RFC in BP and CP of human placentas. A) RFC mRNA was determined by RT-qPCR in relation to the geometric mean expression of 3 housekeeping genes (GAPDH, YWHAZ and β-actin) and expressed as median and 25-75^th^ percentiles. ^#^p = 0.035 *vs*. term newborns, Mann Whitney test. B) Western blots using β-actin as calibrator and Caco2 cells as positive control. C) Western blot signals were determined by densitometry and expressed as median and 25-75^th^ percentiles. RFC: reduced folate carrier; BP: basal plate; CP: chorionic plate.

**Fig 2:**
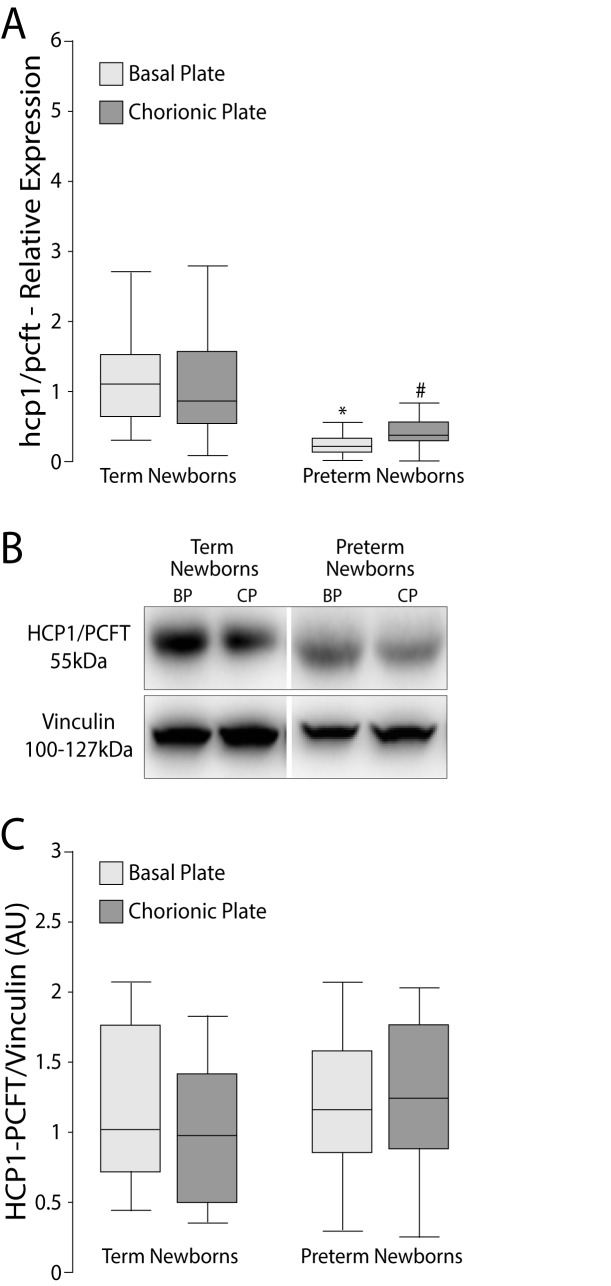
Relative expression (mRNA) and protein levels of PCFT/HCP1 in BP and CP of human placentas. A) mRNA of PCFT/HCP1 was determined by RT-qPCR in relation to the geometric mean expression of 3 housekeeping genes (GAPDH, YWHAZ and β-actin) and expressed as median and 25-75^th^ percentiles. *p<0.0001; # p<0.0001 *vs*. term newborns. B) Western blots using vinculin as calibrator. C) Western blot signals were determined by densitometry and expressed as median and 25-75^th^ percentiles. Mann Whitney test. PCFT/HCP1: proton-coupled folate transporter; BP: basal plate; CP: chorionic plate.

**Fig 3:**
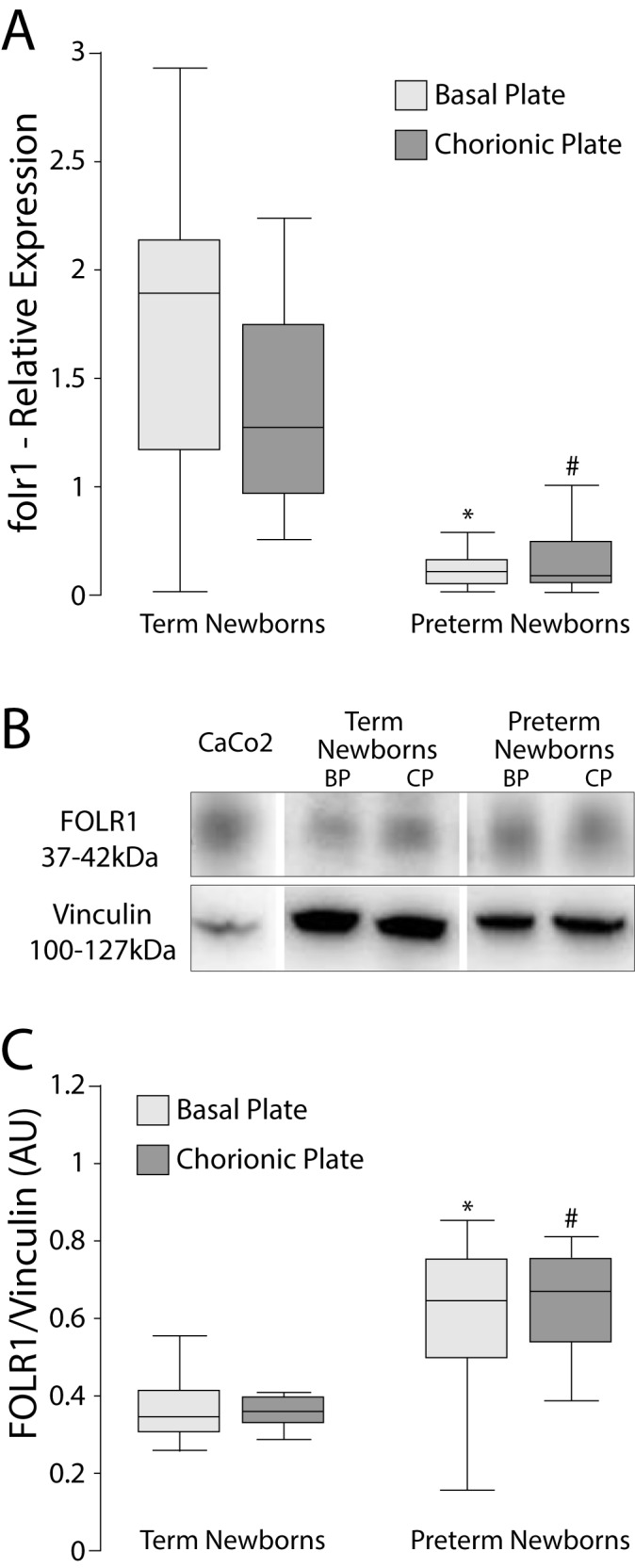
Relative expression (mRNA) and protein levels of FOLR1 in BP and CP of human placentas. A) mRNA of FOLR1 was determined by RT-qPCR in relation to the geometric mean expression of 3 housekeeping genes (GAPDH, YWHAZ and β-actin) and expressed as median and 25-75^th^ percentiles. *p<0.0001; # p<0.0001 *vs*. term newborns. B) Western blots using Vinculin as calibrator and Caco2 cells as positive control. C) Western blot signals were determined by densitometry and expressed as median and 25-75^th^ percentiles. *p <0.0013; ^#^p <0.0001 vs term newborns. Mann Whitney test. FOLR1: α folate receptor; BP: basal plate; CP: chorionic plate.

The mRNA expression of RFC was lower only in the chorionic plate of PT (p = 0.035), **Fig 1A**. The PCFT/HCP1 expression of the PT group in both plates (BP and CP) was lower than the corresponding plate in the term newborns (p <0.0001, both plates **Fig 2A**). In addition, the mRNA expression of FOLR1 in BP and CP of PT placentas were significantly lower than the control group (p = 0.0001 for CP and BP, **Fig 3A**).

### Protein levels of folate transporters

No significant differences were found in protein levels of RFC or PCFT/HCP1 on either placental side (**Figs 1C** and 2**C**). The protein level of FOLR1 in PT placentas was higher in BP (p = <0.0001) and CP (p = 0.0013) compared to the corresponding side of term placentas (**Fig 3C**).

### Concentration of folates and vitamin B12 in cord blood serum

**Fig 4:**
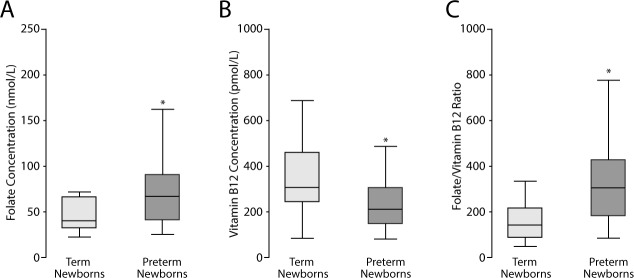
Folates and vitamin B12 concentrations in cord blood serum of newborns. A) Folate concentrations in umbilical cord blood serum are expressed in nmol/L and represent the median and 25-75^th^ percentiles. *p = 0.049 *vs*. term newborns. B) Vitamin B12 concentrations in umbilical cord blood serum are expressed in pmol/L and represent the median and 25-75^th^ percentiles. *p = 0.037 *vs*. term newborns. C) Folate/vitamin B12 ratio is expressed as median and 25-75^th^ percentiles. *p = 0.002 *vs*. term newborns, Mann Whitney test.

The PT group had higher folate concentrations compared to the term group (p = 0.049) (**Fig 4A**). In this group, folate concentrations were 66.4 nmol/L (median) and 41.2–90.4 nmol/L for the 25^th^-75^th^ percentiles (**Fig 4A**). In term newborns, folate concentrations were 40.8 nmol/L (median) and 32.9–66.3 nmol/L for the 25^th^-75^th^ percentiles. PT newborns had lower vitamin B12 concentrations in cord blood compared to the term group (p = 0.0368) (**Fig 4B**). In this group, vitamin B12 concentrations were 209.7 pmol/L (median) and 144.8–304.9 pmol/L for the 25th-75th percentiles. In term newborns, vitamin B12 concentrations were 307 pmol/L (median) and 242.3–458.9 pmol/L for the 25^th^-75^th^ percentiles. PT newborns had a higher folate/vitamin B12 ratio compared to term newborns (p = 0.0017) (**Fig 4C**).

### Correlation analyses

FOLR1 mRNA in CP (Rho = 0.72, p<0.0001) and BP (Rho = 0.71 p<0.0001) was positively correlated with gestational age. Protein levels of FOLR1 were negatively associated with gestational age in CP (Rho = -0.70, p<0.0001) and BP (Rho = -0.43, p = 0.0036). The folate/vitamin B12 ratio was negatively associated with gestational age (Rho = -0.43, p = 0.0002).

## Discussion

In this study we present results indicating that mRNA and protein levels of folate transporters are different in placental tissue of preterm compared to those of term newborns (control), with differences mainly related to their location within the placenta (BP and CP). Similar changes were observed in placentas of term pregnancies with newborns of different birth weight, as recently reported by us [30]. Preterm placentas had lower FOLR1 mRNA than controls, likely due to a negative feedback on the *folr1* gene to prevent further folate transfer to the fetus. In addition, FOLR1 mRNA correlated positively with gestational age. By contrast, FOLR1 protein concentrations were higher in PT than in term placentas, justifying the higher concentrations of folate found in cord blood of PT newborns. In addition, PT newborns had lower vitamin B12 concentrations in cord blood than those found in term newborns.

Previous reports using animal models or cultured cells have demonstrated that folate transporter systems are regulated by changes in extracellular folates [31–34]. Hou et al, (2014), using cultured HeLa cells showed that RFC undergoes posttranscriptional regulation in response to folate excess or deficiency [35]. To our knowledge, our results have shown for the first time that folate concentration in serum cord blood of newborns may be related to the expression of FOLR1 in human placentas.

The placenta is an organ that suffers modifications and adaptations during the course of pregnancy [36,37]. Solanky et al, (2010) reported that placental folate transport is established early in pregnancy, and that folate transporters may change during the course of pregnancy [22]. Some authors have reported that the expression of *RFC* and *PCFT/HCP1* genes decreases between the first and last trimester of pregnancy, while FOLR1 does not change during the gestational period [20,22,38]. This suggests that FOLR1 may be the main regulator for the increased transport of folates from mother to fetus as pregnancy progresses [25]. FOLR1 favors the binding and transport of 5-methyltetrahydrofolic acid (5-MTHF) in a bidirectional way to the fetus against a concentration gradient [3], suggesting that FOLR1 may be the main carrier of folates during the whole pregnancy [24].

Our results show that folate concentrations in cord blood samples are consistent with the public policy of wheat flour FA fortification initiated in Chile in 2001, suggesting that in Chilean pregnant women and their newborns there is no folate deficiency [39]. Folate concentrations in PT newborns observed in the present study are higher than those found in studies conducted in other countries with folate food fortification policies (75^th^ percentile = 79 nmol/L) [40] and without mandatory fortification (95^th^ percentile = 95 nmol/L) [41]. However, in the group of term pregnancies, the 75^th^ percentile of cord blood folate concentration was lower than the levels reported in countries with fortification [40], and the 95^th^ percentile was much higher than that reported in countries without fortification [41]. A weakness of our study is probably the lack of information about maternal folate intake and supplementation during pregnancy. However, and as previously reported, folate concentrations in cord blood may reflect maternal intake, thus we can assume higher intake of folates by mothers delivering newborns with higher cord blood folates [42–44].

Since folate metabolism is highly related with vitamin B12 we also measured their concentrations in cord blood, which was lower in PT than in term newborns. In a Chilean study, two years after FA fortification via wheat flour, 13% of women of childbearing age showed blood vitamin B12 levels below 185 nmol/L, and 10% below 149 nmol/L [45] suggesting that both pregnant women and their newborns may have low levels of vitamin B12. A good marker for vitamin deficiency would have been the measurement of methyl malonic acid (MMA), whose values should have risen under vitamin B12 deficiency [46]. Since vitamin B12 is essential in folate metabolism, the folate/vitamin B12 ratio may be a relevant factor influencing the gestation period.

Although we do not have data on dietary habits and maternal vitamin B12 intake, it has been shown that maternal levels (intake and serum) of vitamin B12 correlate positively with serum levels in newborns [47]. In our study, vitamin B12 concentrations in PT newborns were lower than vitamin B12 concentrations in the control group and also lower than vitamin B12 concentrations reported by Bjørke et al, in cord blood of term newborns [47]. In that report [47] however, vitamin B12 concentrations were similar to those found in the control group of the present study. In addition, the 5^th^ percentile of vitamin B12 concentration in cord blood of PT newborns was lower (97 pmol/L) than the 5^th^ percentile value reported in a Norwegian study (120 pmol/L) [41]. All these results indicate that vitamin B12 concentrations in newborns are variable among different countries.

Actually, there is no consensus regarding optimal folate and vitamin B12 levels in newborn cord blood. Therefore, according to our study we only can establish that a higher folate/vitamin B12 ratio is associated with PT newborns compared to the same ratio in the control group (term newborns). Several studies have shown that folate deficiency and/or an imbalance in the intake of folates and vitamin B12 along with an altered folate/vitamin B12 ratio in blood during and at the end of pregnancy are associated with low birth weight, spontaneous abortion, placental abruption and congenital malformations [48,49].

It is highly likely that epigenetic mechanisms such as alterations in DNA methylation may be involved, leading to an altered expression of certain genes which may affect the offspring phenotype [50,51]. Children at various ages (until 13 years) whose mothers consumed high levels of folates and low of vitamin B12 during gestation had increased risk of developing metabolic diseases such as insulin resistance, obesity and cardiovascular diseases [52,53]. Although there is no evidence to date that high FA intake has negative effects during pregnancy, further studies are necessary to understand the involved mechanisms and possible long-term negative effects [54].

In summary, we found that gestational age is positively associated with placental FOLR1 expression (mRNA) and negatively with folate/vitamin B12 ratio in cord blood serum. Our results clearly suggest that in PT newborns, placental FOLR1 plays a main role in ensuring an adequate transport of folate to the fetus, resulting in higher folate concentrations, and as a consequence an increased folate/vitamin B12 ratio in PT newborns cord blood.
